# Podcasting and Blogging as Tools to Engage with the Public on the Topic of Cancer: Experience and Perspectives of the Public Interest Group on Cancer Research

**DOI:** 10.3390/curroncol32100579

**Published:** 2025-10-18

**Authors:** Sevtap Savas, Kayla Crichton, Jason Wiseman, Janine Taylor-Cutting, Tracy Slaney

**Affiliations:** 1 Division of Biomedical Sciences, Faculty of Medicine, Memorial University, St. John’s, NL A1B 3V6, Canada; kcrichton@mun.ca; 2 Public Interest Group on Cancer Research, St. John’s, NL A1B 3V6, Canada; 3 Division of Population Health and Applied Health Sciences, Faculty of Medicine, Memorial University, St. John’s, NL A1B 3V6, Canada; 4 Discipline of Oncology, Faculty of Medicine, Memorial University, St. John’s, NL A1B 3V6, Canada

**Keywords:** neoplasm, blogging, Newfoundland and Labrador, patient participation, stakeholder participation, webcast

## Abstract

**Simple Summary:**

Our group launched a podcast and guest blog series in 2024 to communicate knowledge about cancer, including lived experiences of cancer, clinical care, research, advocacy, and public engagement. In this paper, we reflect on our experience with this podcast and guest blog series. With the help of 36 guests, we generated 13 blogs and 28 podcast episodes in a remarkably cost-effective way. Generally, this experience was positive for both our team and our guests. However, we also encountered barriers—notably, challenges related to guest compensation. For example, in some cases, guest compensation was not possible because of federal or university policies or practices. Despite these challenges, we used our learnings to improve our and guests’ experience whenever possible. In conclusion, our model may offer guidance to other public-academic partnerships and knowledge mobilization efforts. Crucially, governments and institutions must do more to ensure equitable participation by enabling appropriate compensation for all contributors.

**Abstract:**

We (Public Interest Group on Cancer Research) started a podcast and guest blog series on cancer in 2024. Our objective in this Commentary is to describe our experience with this series, insights gained, adjustments made to our approach, and our recommendations for future series. Our group identified and invited guests to contribute a blog or podcast episode on cancer, lived experience of cancer, cancer care and research, or advocacy. The podcast episodes were recorded using the WebEx platform (version 45.9.0.33069) and edited using the Kdenlive software (version 23.08.4). The blogs and podcasts were edited, finalized, and posted online for public access. In this manuscript, we utilized descriptive statistics to define and summarize information about the podcast episodes, guest blogs, and categorical responses to guest feedback survey questions, while we presented the responses to open-ended survey questions as quotes and summaries. As a result, during the period of January 2024–July 2025, we aired 28 podcast episodes and 13 guest blogs involving 36 guests. Guests included people from various backgrounds (such as people with lived experience, advocates, scientists, and healthcare providers) and members of equity-deserving communities (such as women, Indigenous and 2SLGBTQIA+ communities). We contemplated and learned as we proceeded with this project and implemented changes to address the issues that arose. In most cases the guests had positive experiences; however, in rare cases, university practices or federal policies prevented guest compensation, creating an unusual barrier. In conclusion, podcasting and blogging are practical public engagement instruments that provide space for sharing messages and knowledge to communicate with members of the public. Systematic barriers, such as policies that hamper guest compensation, need to be fixed for equitable participation, compensation, and engagement. As there is an increased interest in public engagement and knowledge mobilization activities, our learnings shared in this commentary may help other groups initiate or improve their public engagement practices.

## 1. Introduction

Active participation of the very people affected by a condition or issue can improve project design and conduct, interpretation and dissemination of results, and putting the project-generated knowledge into action.

As a result, there is a strong emphasis on public and patient engagement and partnership in health research [1,2,3,4]. Knowledge mobilization is tightly linked to engagement, aiming to disseminate and utilize knowledge and hence positively impact the society (and/or the systems that serve the society) [5,6,7,8,9]. In Canada, the strategy for patient-oriented research (SPOR), the support provided by several SPOR units across the country, and organizations such as Research Impact Canada have been instrumental in strengthening public engagement and knowledge mobilization practices [1,5,10].

Among the digital tools that can connect, engage, communicate, mobilize and exchange knowledge with the public are blogs and podcasts. Podcasts are increasingly used as public platforms to communicate knowledge and experiences about cancer. Examples include international, national and local health-related organizations, institutions, community support organizations, and personal podcasts (e.g., [11,12,13,14]). Personal and organizational blogs on cancer are also abundant (e.g., [15,16]). These digital resources aim to disseminate conversations widely and in a flexible delivery mode (e.g., podcast recordings and blogs are suitable for being accessed at a time and place that is good for the user).

A recent systematic review [17] found that podcasts can be beneficial, feasible, and flexible tools in patient health-related education, while also noting potential barriers to access, such as availability of the internet and device—this barrier is also applicable to blogs posted online. By providing space to those who would like to share their stories, opinions, perspectives, experiences, or expertise, and by being freely accessible on the internet, these digital tools may inform interested individuals and organizations, initiate public discussions, and elevate the voices of people with lived experiences (PWLE). They can also be used as effective tools for delivering scientific and medical knowledge by experts—this may be particularly beneficial considering the widespread presence of misinformation and disinformation that can cloud the individual judgment and damage the public’s trust in science and public health policies [18,19,20].

Our work previously identified that there was a need to inform and educate the residents regarding cancer [21]. Considering the fact that a local blog and podcast series on cancer-related topics (which was informed and co-led by public members) would help address this need, in 2024, our group started a project to create and disseminate digital outputs by focusing on guest blogging and podcasting on cancer. Our group (The Public Interest Group on Cancer Research [PI Group]) [21] is a public-scientist partnership in Newfoundland and Labrador (NL), a Canadian province with one of the highest cancer incidence and mortality rates in the country [22]. The blogging and podcast project was funded for the second year in early 2024, currently leading to two Seasons. During this time, the PI Group co-produced podcast episodes and guest blogs on cancer with PWLE, advocates, researchers, clinicians, and leaders.

In this manuscript, we describe our experience with leading the creation of these digital contents, satisfaction of and barriers experienced by guests as well as our team, and recommendations for better and more effective public engagement and outreach strategies using blogging and podcasting.

## 2. Materials and Methods

### 2.1. Definitions

Guest blogs refer to written content that is created by guest contributors and posted on our website for public access.

Podcast episodes refer to sound recordings involving conversations between at least one host and one guest speaker that are posted online for public access.

### 2.2. Generation of Project Idea, and Identification of Topics and Guests

The idea of creating a podcast where our group would act as the host and invite guest speakers to have conversations about cancer-related topics was brought to the table by a PI Group member (JW). Guest blogs as a second form of digital output were added, as they provide flexibility (e.g., guests can write their blogs at a time and manner that they choose, and they have the option of being anonymous). A third option of posting a video recording of the conversations was exercised only once—this recording was posted as an MP4 file on YouTube [23] and as an MP3 file on Spotify (Episode 3).

Most topics and guests of interest have been identified by the PI Group during its virtual meetings (2023–2025) and were noted in meeting minutes. The Group leader (SS) then invited guests. In rare cases, requests came from guests, which were honoured. The PI Group members were invited to participate in the creation of the podcasts and blogs.

All guests gave permission to make their digital assets publicly available on the internet. Information about the project and sample questions was also provided for guidance.

This project was co-led by a public member/PWLE (JW) and a scientist (SS).

### 2.3. Creation of Guest Blogs and Podcast Episodes

The guest bloggers had the option of being anonymous. In two cases, long writings were divided into pieces and posted as multiple blogs. The guest blogs were edited by the project leader (SS) and posted on the PI Group’s website [24]. The blogs by the guest from Turkiye were initially written in Turkish, which were then translated by an online translator [25], and corrected and verified by the project lead (SS), who is bilingual.

Podcasting was a new activity for the PI Group. The creation and tech-related features of the podcast have been described earlier [26]. In brief, podcast episodes were conducted as WebEx meetings, a platform vetted by Memorial University, and recorded. The video file (MP4) was then converted into a sound file (MP3). Kdenlive software [27] was used for this purpose and to edit and finalize the podcast episodes. Each podcast episode was accompanied by a short description and posted on Spotify [28]. Most podcast episodes were hosted by the project leader. In co-hosted podcast episodes, the co-hosts were members of the core podcast team (select members of the Public Interest Group [JW, JT-C, TS], and the project assistant [KC]). Two of us acted as the guest and cohost in separate episodes (TS, SS).

**Figure 1:**
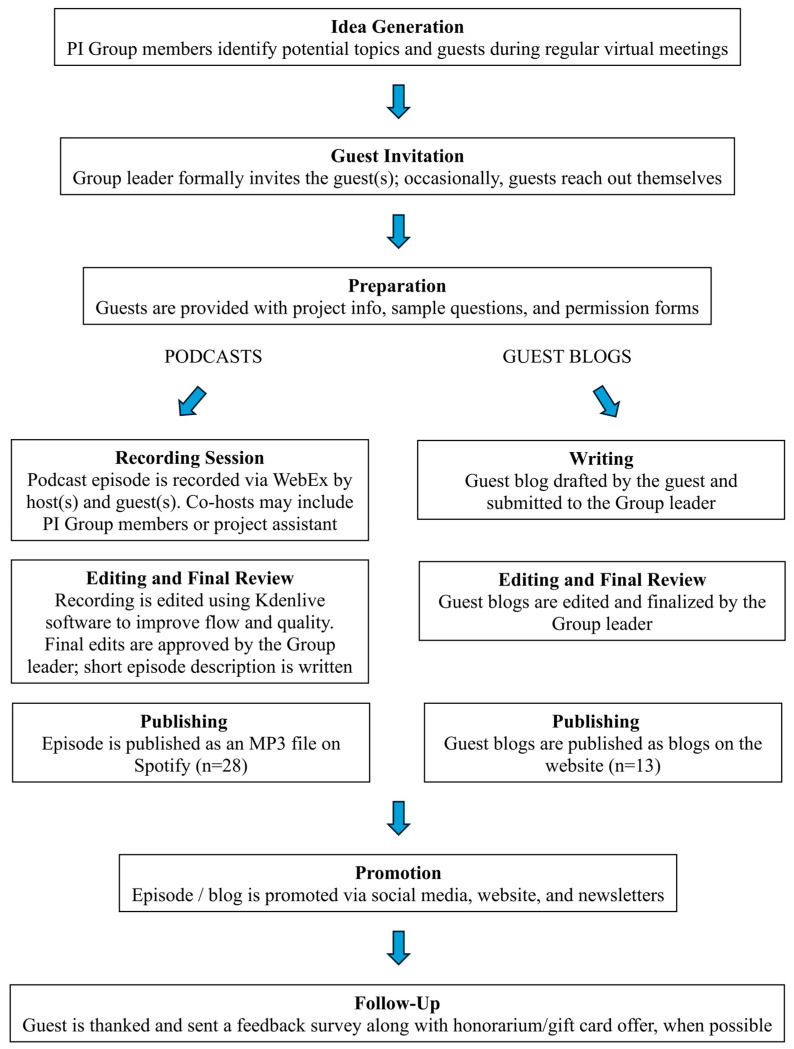
Flowchart depicting the key steps of the project. PI Group: Public Interest Group on Cancer Research.

Figure 1 summarizes the key processes undertaken to create the blogs and podcast episodes.

Our project team implemented measures to help prevent sharing misinformation and disinformation. The podcast guests included several scientists and healthcare providers—they were invited specifically to provide a solid scientific and medical background to the public via this project. In addition, podcast questions were drafted and shared with the guests before recording, which targeted specific topics. Last, the project lead, who is a cancer scientist (SS) and edited the guest blogs and podcasts, reviewed the contents to make sure that they would not include misinformation or questionable practices/information.

### 2.4. Promotion of Guest Blogs and Podcast Episodes

Social media and website posts were the primary ways used to increase awareness and access to podcast episodes and guest blogs. We also promoted these outputs through recaps and the NLSUPPORT newsletter [29,30,31,32]. Conference presentations (CAPO 2025 annual conference, Engage Memorial Symposium 2025) were utilized to promote and increase awareness about the project and its outputs within the academic environments.

### 2.5. Obtaining and Summarizing Feedback from the Guest Contributors

A Qualtrics survey was created to receive feedback about the experiences of the guest bloggers and podcasters. The link to the survey was emailed to the guests after their contribution was uploaded on the website (blogs) or aired on Spotify (podcasts). A reminder email was sent a week later. The categorical responses were summarized by descriptive statistics. Responses to open-ended questions were summarized, with some of them also being integrated here as quotes. A copy of the survey can be found in Supplementary Information—Table S1.

### 2.6. Compensation of Guest Contributors

As per the CIHR’s recommendations [33], we have offered compensation to guest bloggers ($25 honorarium in Season I, $100 honorarium or gift card in Season II) and podcast guests ($100 honorarium in Season I, $100 honorarium or gift card in Season II). Priority given to the public member guests, considering the fixed funding and the co-production of more digital content than planned. Compensation was not offered in cases of perceived conflict of interest (i.e., when the guest is a family member of a project team member). The PI Group members were also offered an honorarium/gift card annually for their membership and participation in the PI Group activities.

### 2.7. GRIPP2 Checklist

The GRIPP2 checklist document can be found in Supplementary Information—Table S2.

## 3. Results

### 3.1. Our Experience with Creating This Podcast and Guest Blog Series

#### 3.1.1. Guest Blogs and Podcast Episodes

In two Seasons, a total of 41 digital outputs were created: 13 guest blogs and 28 podcast episodes (Supplementary Information—Table S3).

In both Seasons, the first podcast episode was aired on the day of February 4th to commemorate World Cancer Day, as per the suggestion of a PI Group member.

The length of the podcast episodes varied between ~28 min to 1 h 22 min. Episodes were aired on predictable dates of months (4th, 17th, and occasionally 24th and 27th).

Guest blogs were made public in a less scheduled way, and their post dates were spaced at least a week apart when possible.

#### 3.1.2. Guest Creators and Podcast Co-Hosts Involved in the Project

A total of 40 people were involved as guests (*n* = 36) and co-hosts (*n* = 5; two hosts also acted as guests). The guests included PWLE (21 guests in 30 outputs), leaders and champions (e.g., leaders of community support organizations, advocates, fundraisers, some of whom were also PWLE; *n* = 13 outputs), researchers (*n* = 5 outputs), and healthcare providers (*n* = 6 outputs) (Supplementary Information—Table S3).

While Season I focused on local context with only three guests from outside of the province, Season II aimed to increase diversity of perspectives, and hence, purposefully invited guests from other Canadian provinces and countries. Overall, most guest bloggers were from Canada, while two bloggers were from Turkiye and England. In terms of the podcast, seven episodes had guests from other Canadian provinces or Territories (British Columbia, Prince Edward Island, Nova Scotia, Quebec, and the Northwest Territories). Overall, guests from outside of the province constituted 10/36 of the guests (28%). Blogs and podcast episodes that included guests from outside of the province constituted 15/41 of the contributions (37%).

Equity-deserving communities, such as racialized minorities and Indigenous and 2SLGBTQIA+ community members, were represented in (at least) six outputs. While most podcast episodes had one host, two co-hosts led the conversation in eight episodes. In six episodes, two guests spoke with the host(s).

#### 3.1.3. Topics Covered in the Digital Products

**Figure 2:**
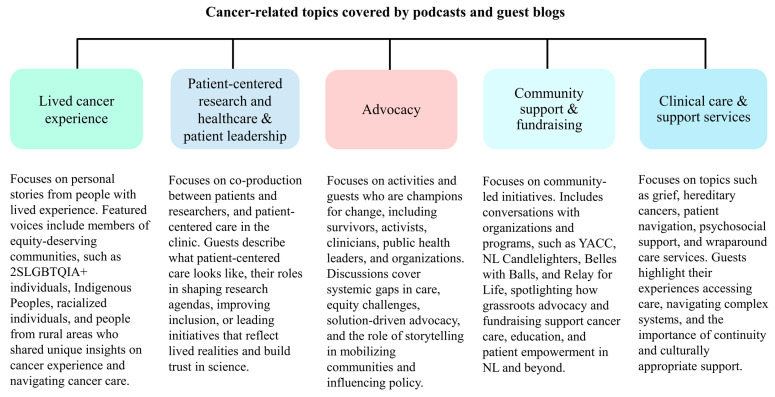
Podcast and guest blog contents. CCS: Canadian Cancer Society, NL: Newfoundland and Labrador, YACC: Young Adult Cancer Canada.

All blogs were on lived cancer experience, and a variety of topics around cancer were covered in the podcast (Figure 2).

Two of the three most accessed podcast episodes included the guest (James Moriarty), a transgender man with a lived experience of cancer.

#### 3.1.4. Cost Effectiveness of the Project

The total funding cost, including compensation, headsets bought (for co-hosts), and tech/administrative support by the assistant, was approximately $20,000. The series, however, required considerable time commitment by the project lead and had no specific funds allocated for promotion.

### 3.2. Satisfaction of and Barriers Experienced by the Guests and Our Team

#### 3.2.1. Satisfaction of and Barriers Experienced by the Guests

Across Seasons I and II of the podcast and blog series, guest contributors consistently reported highly positive experiences. The vast majority agreed that their contributions were valued, the process was smooth, and they were satisfied with the final product. Contributors noted that hosts were excellent and scheduling was easy, with many respondents expressing gratitude for the opportunity to participate.

Despite challenges, such as administrative barriers to international compensation (*see below*), most guests described their involvement as meaningful, empowering, and enjoyable, reflecting strong engagement and a supportive environment. See Table 1 for a selection of guest quotes that highlight the positive guest experiences with the podcast and blog series, meaningful engagement, connection, appreciation, and overall satisfaction across both Seasons.

#### 3.2.2. Satisfaction of and Barriers Experienced by Our Team, Insights Gained, and New Practices Implemented

We used the lessons learned in Season I to make Season II better, while also experiencing new issues in Season II.

##### Better Organization and Lowered Burden

Our take on Season I of the podcast, particularly on the scheduling issues we experienced, can be found in an earlier publication [26].

In Season II, we were better organized and started podcast recordings early (November 2024) and completed all episodes before May so that they could be posted by the end of July. Starting early also helped us with having a longer period to record the podcasts and hence, not taxing the host(s). Season II also included fewer podcast episodes than Season I, giving our team some time relief.

##### Compensation Issues

There were two different types of compensation issues we experienced in Season I and II.

In Season I, we were not able to provide honorarium to a transgender guest because their current name did not match their dead name associated with their social insurance number (SIN). Luckily, we solved this issue with the help of the funder, who approved providing gift cards to this guest. This experience prompted us to formally include gift cards (not only honorarium) in Season II budget as a form of compensation.

However, in Season II, we experienced a different type of compensation issue. Our finance office was not able to provide a gift card to an overseas guest that can be used in their country or transfer the honorarium funds to their country via banking (likely fueled by limited ways to honour compensation as per university policy, involvement of different staff and/or lost information in email threads). Consequently, this guest was very dissatisfied and disappointed. As a result, we lost the opportunity to collaborate with this important voice in future projects.

These incidents underscore how existing compensation systems often exclude the very people that public engagement efforts seek to include. Addressing these barriers is not just an administrative task—it is an ethical imperative.

##### Increased Diversity of Voices

We found that increased interactions with national and international guests in Season II were beneficial as they helped us connect with new people and see what common and unique experiences are shared within local, regional, or global contexts. It also helped us promote the PI Group widely.

We felt that having healthcare providers as podcast guests was more challenging than other types of guests. One reason for this could be the need to get permission for healthcare providers to speak from their organization (in one case, we had to postpone the podcast recording so that one of our guests could get this permission). This is consistent with our previous experience working with healthcare providers.

##### Access to Outputs

Promotion of the podcasts and guest blogs takes time and happens over time. Overall, we thought that the access to the generated outputs needed to be improved. For example, as of 4 October 2025, the total numbers of streams and downloads and Spotify plays were 438 and 405, respectively.

##### Our and Guests’ Growth

Last, this has been a great experience for the PI Group members involved in podcasting and blogging. Hence, we built new skills by co-creating/creating blogs and podcasts, editing podcast episodes, and co-hosting episodes. We also had fun during the podcast, formed deeper connections and relations with the public and guests, were generally satisfied with what we did, and wished that more people used it as a tool.

We also believe that skill building was a part of the guest experience (many of the guest bloggers and podcast guests probably had not done these activities before).

## 4. Discussion

In this commentary, we describe our experiences with having a podcast and guest blogging series on cancer, a public engagement project co-created and co-led by public members and scientists. Our experience shows that both of these public engagement and knowledge mobilization tools are cost-effective, provide space for those who are willing to share their stories, expertise, or advocacy, and are often perceived as a positive experience by the contributors/guests. Our experience also shows that sometimes, institutional policies and practices place extra barriers to participation and compensation and further deepen the inequity.

We found the barriers to compensation the most disturbing. In a time in history, where we prioritize removing barriers and addressing inequity, it is disappointing that the government, funding, or institutional policies are a part of the problem themselves. We call for the federal government to remove the name-matching barrier for transgender individuals while honouring honoraria and Memorial University (and other universities) to fix financial service policies and practices to provide timely compensation to overseas and transgender honorees.

This project has been a product of a key mandate of the Public Interest Group on Cancer Research, which is to engage with the public and exchange knowledge on cancer in Newfoundland and Labrador [21]. This Canadian province has one of the highest cancer rates in the country, making cancer a serious health and healthcare concern with a significant impact [22]. The cancer care is provided by the Provincial Cancer Care Program and includes prevention (e.g., smoking cessation program), treatment, support services (e.g., oncology social work, patient navigator program), and screening programs (e.g., cervical, colon, and breast cancer screening programs) [34]. Despite these services being offered and dedicated staff, the public uptake of these programs does not appear to be high or effective [21,35]. As a result, organizations, such as the Canadian Cancer Society [36], Young Adult Cancer Canada [37], NL Candlelighters (focusing on pediatric cancers in NL [38]), Belles with Balls (focusing on ovarian cancer in NL [39]), advocates, and groups like ours carry the obligation to connect with the community and engage them in conversations that can be influential in cancer prevention, early detection, treatment or support.

However, as we expressed in other venues as well [40], we emphasize that the real responsibility belongs to the health care system and governments. Therefore, we once again call for public information campaigns that will be effective, long-lasting, and comprehensively address the information needs of the population to help control cancer. We also ask healthcare systems to allow healthcare providers to speak more freely and frequently in public and media settings to provide communities with reliable medical knowledge and information and help address health-related misinformation at the population level.

As one PI Group member said, *there are a lot of resources out there*. We contemplated this comment and our overall experience as co-creators of the digital outputs. While we benefited from doing this series (in terms of connecting with new people, learning from guests, communicating lived experience and scientific-medical knowledge, having fun and building on our skills or developing new interests) and it is a cost-effective way to achieve our goals, we are now inclined towards reducing co-creation and increasing promotion of the outputs generated as a result of this project. We also think that promotion (for example, through social and printed/digital media ads) should be a part of any public engagement, scientific communication, or knowledge mobilization plan, and as such, proposers and funders should consider promotion-related expenses as an integral part of the applications.

While scholarly papers about experiences with podcasting and blogging on cancer are rare, a recent publication on a podcast-based initiative about radiation treatment and young adult cancer patients [41] reported similar experiences to ours. For example, the authors stated the need for flexibility and adjustments, value of and satisfaction with conversations, acquiring new skills and/or knowledge during the process, and continuation of relationships between the hosts/guests after the podcast. Our collective knowledge in this evolving area will increase as more authors share their experiences.

We acknowledge the benefits of continuous learning in our approach to public engagement. By observing, conversing among us, and paying attention to the feedback received, we were able to detect issues and address some of them by making the necessary adjustments in our project. For example, during Season I, we worked on our scheduling issues [26]. Better organization and lowering the burden of podcasts in Season II lowered our overall stress and time commitment. Adding the option of gift cards in Season II as a means of compensation removed one barrier to equal compensation. However, we are yet to find a solution to compensate individuals living overseas if institutional policies do not change anytime soon. The only viable option seems to be the gifts provided by the project team from their own pockets.

Limitations of this public engagement project should also be mentioned. They include English being the only language used in the guest blogs and podcast episodes (while both English and French are the official languages of Canada), public outreach being relatively limited (due to lack of specific funding for promotion purposes), not having all relevant topics covered (due to resource and time limitations), and not having all undeserved communities represented by the guests (such as the Black community).

## 5. Conclusions

From lived experiences to cancer care, there is something for everyone in the outputs generated during this project—we invite everyone to have a look at them. Because of their digital nature, these series can be accessed by anyone freely, including those in rural and remote areas. We believe that our experience, perspectives, contemplations, and feedback received and described in this article may inform other partnerships and researchers who would like to use blogs and/or podcasts as tools of public engagement, science communications, and knowledge mobilization. Future public engagement activities could include interactive approaches (such as virtual townhalls), accessible public knowledge sessions (such as virtual sessions, courses, and programs), and increased representation by the underserved communities. The issues regarding compensation described here serve as a reminder that governments, funders, and institutions can unintentionally create barriers and inequities through their own policies—now is the time to address and rectify these.

## Figures and Tables

**Table 1:** Guest quotes from feedback surveys (Seasons I and II).

Quote
“Thank you so much for this opportunity, I had a great time sharing my experience and felt really heard.”
“My comment is that I think this podcast platform is incredibly important for sharing patient and caregiver voices. It allows for meaningful connection.”
“It was a great experience and I’d recommend it to others.”
“Great session and really enjoyed the conversation.”
“Thank you so much for the thrilling opportunity!”
“I just love this group.”
“I believe sharing my experiences/feelings as a cancer patient allowed to send important messages to the world directly”
“The tremendous air of inclusion, cooperation and collaboration provided by [the host]”

Select quotes by guests who contributed to the series.

## Data Availability

This manuscript summarizes the work of a group of people regarding their experiences with leading a podcast and guest blogging series, and the feedback received. As such, there is no research data. All data generated or analyzed during this work are included in this article and its Supplementary Information Files.

## Supplementary Materials

The following supporting information can be downloaded at https://www.mdpi.com/article/10.3390/curroncol32100579/s1, Table S1. Feedback survey. Table S2. The GRIPP2 checklist. Table S3. Information about the guests and links to their blogs and podcast episodes.

## Abbreviations

The following abbreviations are used in this manuscript:

CIHR	Canadian Institutes of Health Research
NL	Newfoundland and Labrador
NLSUPPORT	Newfoundland and Labrador SPOR and Trials Support Unit
PI Group	Public Interest Group on Cancer Research
PWLE	People with Lived Experience
SIN	Social Insurance Number
SPOR	Strategy for Patient-Oriented Research
