# Supplementary material for: Podcasting and Blogging as Tools to Engage with the Public on the Topic of Cancer: Experience and Perspectives of the Public Interest Group on Cancer Research
Source: Curr Oncol. 2025 Oct 18;32(10):579. doi: 10.3390/curroncol32100579 (PMC12563759; doi:10.3390/curroncol32100579)
Supplement: Supplementary file 1 [file curroncol-32-00579-s001.zip › curroncol-3887323-supplementary.pdf]

Supplementary information

Podcasting and Blogging as Tools to Engage with the Public on the Topic of Cancer: Experience and Perspectives of the Public Interest Group on Cancer Research

Sevtap Savas, Kayla Crichton, Jason Wiseman, Janine Taylor-Cutting and Tracy Slaney

Supplementary information - Table S1. Feedback survey.

Which contribution (asset) have you made for this project (check all that apply)?

- Podcast
- Guest blog

1. Please tell us how much you agree with the following statements

I feel that my contribution (asset) was valuable

I feel that my contribution (asset) was valued by the organizers

The entire process of creating my contribution (asset) was feasible

I was satisfied with the final product (asset)

Information provided prior to creating my contribution (asset) was useful

I was able to get support when I need while creating my contribution (asset)

I would work with this group for similar purposes again

2. In your opinion, what were the MOST VALUABLE aspects of participating in this project / creating your contributions? *(open-ended question)*

3. In your opinion, WHAT CAN BE DONE to further improve this project next time? *(open-ended question)*

4. What OTHER TOPICS would you like to be included in the future projects? *(open-ended question)*

5. Please rate the following
a. Virtual platform/environment

b. Hosts /organizers that I have interacted with

c. Ease of scheduling

d. Ease of creating my contributions (asset)

e. Overall satisfaction with this experience - Selected Choice

6. If you have any other comments, please feel free to tell us in the space below *(open-ended question)*

7. How did you hear about this event? Please check all that apply

- Memorial University communications
- Social media (Twitter, Facebook, etc.)
- Main stream media (radio, TV, newspaper, etc.)
- I was invited
- Other (please specify)

Supplementary information - Table S2. The GRIPP2 checklist.

**Section and topic**	**Item**	**Reported on page No**
1: Aim	Report the aim of PPI in the study	2-3
2: Methods	Provide a clear description of the methods used for PPI in the study	3-5
3: Study results	Outcomes—Report the results of PPI in the study, including both positive and negative outcomes	4-9
4: Discussion and conclusions	Outcomes—Comment on the extent to which PPI influenced the study overall. Describe positive and negative effects	9-10
5: Reflections/critical perspective	Comment critically on the study, reflecting on the things that went well and those that did not, so others can learn from this experience	4-10

PPI*:* patient and public involvement

*Downloaded from: https://researchinvolvement.biomedcentral.com/articles/10.1186/s40900-017-0062-2.

Supplementary Information - Table S3. Blogs and podcast episodes.

a) Blog posts

Name of the guest	Location of the guest	Title of digital output	Link to digital output
		**SEASON I (5 guest blogs)**	
Jenna Neville (PWLE)	Newfoundland and Labrador, CANADA	Leaving our lives in the hands of time	https://strength-in-community.ca/2024/01/24/guest-blog-leaving-our-lives-in-the-hands-of-time/
Deanna Roy (PWLE)	Newfoundland and Labrador, CANADA	A message of hope and healing	https://strength-in-community.ca/2024/01/30/guest-blog-a-message-of-hope-and-healing/
Darrell Peddle (PWLE)	Newfoundland and Labrador, CANADA	Dad would be so proud! (PART I)	https://strength-in-community.ca/2024/02/08/guest-blog-dad-would-be-so-proud-part-i/
Darrell Peddle (PWLE)	Newfoundland and Labrador, CANADA	Dad would be so proud! (PART II)	https://strength-in-community.ca/2024/02/20/guest-blog-dad-would-be-so-proud-part-ii/
Namiko Sakamoto (PWLE)	Newfoundland and Labrador, CANADA	Then there was hope	https://strength-in-community.ca/2024/03/21/guest-blog-then-there-was-hope/
**SEASON II (8 guest blogs)**			
Mehtap Savas (PWLE)	Ankara, TURKIYE	My story of surviving stomach cancer in Turkiye (PART I – INTRODUCTION)	https://strength-in-community.ca/2025/01/13/guest-blog-my-story-of-surviving-stomach-cancer-in-turkiye/
Mehtap Savas (PWLE)	Ankara, TURKIYE	My story of surviving stomach cancer in Turkiye (PART II – LIFE WITH CANCER AND TREATMENT)	https://strength-in-community.ca/2025/01/20/guest-blog-my-story-of-surviving-stomach-cancer-in-turkiye-part-ii/

Mehtap Savas (PWLE)	Ankara, TURKIYE	My story of surviving stomach cancer in Turkiye<br>(PART III – LIFE WITHOUT A STOMACH)	https://strength-in-community.ca/2025/01/27/guest-blog-my-story-of-surviving-stomach-cancer-in-turkiye-part-iii/
Mehtap Savas (PWLE)	Ankara, TURKIYE	My story of surviving stomach cancer in Turkiye<br>(PART IV – RETURN TO WORK AND IMPACT OF CANCER)	https://strength-in-community.ca/2025/02/03/guest-blog-my-story-of-surviving-stomach-cancer-in-turkiye-part-iv/
Mehtap Savas (PWLE)	Ankara, TURKIYE	My story of surviving stomach cancer in Turkiye<br>(PART V – FOLLOW UP: GOOD DOCTORS, BAD DOCTORS)	https://strength-in-community.ca/2025/02/10/guest-blog-my-story-of-surviving-stomach-cancer-in-turkiye-part-v/
Mehtap Savas (PWLE)	Ankara, TURKIYE	My story of surviving stomach cancer in Turkiye<br>(PART VI – CURRENT STATE & FINAL WORDS)	https://strength-in-community.ca/2025/02/17/guest-blog-my-story-of-surviving-stomach-cancer-in-turkiye-part-vi/
Mehtap Savas (PWLE)	Ankara, TURKIYE	My story of surviving stomach cancer in Turkiye<br>(PART VII – SILENCE OF THE MOTHERS)	https://strength-in-community.ca/2025/02/24/guest-blog-silence-of-the-mothers/
John Dabell (PWLE)	ENGLAND	Cancer: Make a good nuisance of yourself	https://strength-in-community.ca/2025/03/03/guest-blog-cancer-make-a-good-nuisance-of-yourself/

PWLE: people with lived experience (individuals affected by cancer directly or indirectly).

b) Podcast episodes

**Name of the guests (hosts)**	**Location of the guests**	**Title of digital output**	**Link to digital output**
**SEASON I (16 podcast episodes)**			
Dr. Sevtap Savas (S)<br><br>(Jason Wiseman, Janine Taylor-Cutting)	Newfoundland and Labrador, CANADA	Episode 1 – A cozy chat about cancer, Newfoundland and Labrador, and fate of a bagel	https://open.spotify.com/episode/3TUG57h8Ks7PMvJuPBYIKm?si=NYWRXy3QTTmb0qObzlDSow
James Moriarty (PWLE)<br><br>(Dr. Sevtap Savas)	Newfoundland and Labrador, CANADA	Episode 2 – At the intersection of being trans and having gendered cancer: A candid, timely, and educational conversation with James Moriarty	https://open.spotify.com/episode/0Bm064SjFMrmryDYTvU5Ck?si=WrPVAvlyQYmdg-Eb6hmQrQ
Derrick Bishop (PWLE), Dr. Jane Green (S)<br><br>(Dr. Sevtap Savas)	Newfoundland and Labrador, CANADA	Episode 3 – "You saved my life": A story of a Newfoundland and Labrador family, genes & cancer by Derrick Bishop and Dr. Jane Green	https://open.spotify.com/episode/1PsR5JFmn2ka13u0MTwN0h?si=iMztaktPRBeCSopHlgVjIA
Sophia Ryan (L/A) and Emma McIsaac (L/A)	Newfoundland and Labrador, CANADA	Episode 4 – “There is a future where we do not have to worry about cancer”: Vision by two young	https://open.spotify.com/episode/6QoqLa0En02HE2H8pQW64y?si=6tyUHTweQneLuGvngoBOSg

(Dr. Sevtap Savas, Kayla Crichton)		leaders - Sophia Ryan and Emma McIsaac	
Geoff Eaton (PWLE; L/A)<br><br>(Dr. Sevtap Savas)	Newfoundland and Labrador, CANADA	Episode 5 –"Prepare to die, but plan to live": An impactful chat with Geoff Eaton about his lived experience, young adult cancers, and Young Adult Cancer Canada (YACC)	https://open.spotify.com/episode/4DThIQq2HJpS8Y4LnJqYN5?si=jQs7qoMOTDyRAhYUmFur5A
Alan Winnett (PWLE; L/A)<br><br>(Dr. Sevtap Savas; Jason Wiseman)	Newfoundland and Labrador, CANADA	Episode 6 – A story of childhood cancers, resilience, and using lived experience to support others– Alan Winnett from Candlelighters NL shares	https://open.spotify.com/episode/0Oa6GMSIcK3kSk01DvQtX5?si=MiAiArvmS2yazM4v6kHHWQ
Dr. Maisam Najafizada (S)<br><br>(Dr. Sevtap Savas)	Newfoundland and Labrador, CANADA	Episode 7 – Do you get patient-centered cancer care? A cozy, hopeful, and inspirational chat with Dr. Maisam Najafizada	https://open.spotify.com/episode/1iXHFMczyhlnhCLnPZWToy?si=S1nIcj9XSjeKFKKBtUHwDg
Bonnie Morgan (PWLE; L/A), Alana Walsh Giovannini (PWLE; L/A)<br><br>(Dr. Sevtap Savas)	Newfoundland and Labrador, CANADA	Episode 8 – "I had a tumor of the size of a tissue box" –Bonnie Morgan and Alana Walsh-Giovannini tell us about ovarian cancer and Belles with Balls	https://open.spotify.com/episode/4WscyYHSW21pY5IsHM8O3v?si=dswyA-zLRPqy53-cFiDf4A

Stephanie Budgell (PWLE)<br><br>(Dr. Sevtap Savas; Janine Taylor-Cutting)	Newfoundland and Labrador, CANADA	Episode 9 – Those with lived experience are the best teachers and speakers: Listen to our chat with Stephanie Budgell	https://open.spotify.com/episode/5I403W7f2rfELQnfDKJ9VC?si=FAdNuICcRJ-AX299F5o9wQ
James Moriarty (PWLE) and Dr. Joannie Neveu (HCP)<br><br>(Dr. Sevtap Savas)	Newfoundland and Labrador, CANADA	Episode 10 – At the intersection of being trans and having gendered cancer - PART II: Interview with James Moriarty and Dr. Joannie Neveu	https://open.spotify.com/episode/0Ye4HFd7h4UdVq8n1NUrvE?si=qR89QaEkSkC72M06bAO81Q
Tracy Slaney (PWLE; L/A)<br><br>(Dr. Sevtap Savas)	Newfoundland and Labrador, CANADA	Episode 11 – Push For Your Tush! Listen to Tracy to hear how her family is living with cancer but not ruled by it	https://open.spotify.com/episode/76piiGiwItKCHiB5GMkJO9?si=otBZO1XDTb2DFZ8oTDaZSA
Ashley Dicker (HCP)<br><br>(Dr. Sevtap Savas)	Newfoundland and Labrador, CANADA	Episode 12 – Indigenous Patient Navigator Program in Newfoundland and Labrador – Ashley Dicker shares important knowledge about this impactful healthcare service	https://open.spotify.com/episode/1tETVhV2oGTj0UXZKarZCi?si=gBrARRjZQzedWwLmK0MGQg
Dr. Robin McGee (PWLE; L/A), Hope Gillis (HCP)	Nova Scotia, CANADA	Episode 13 – “I have led people to the promised land, but I could not enter the promised land”. In Memory of Dr. Robin McGee	https://open.spotify.com/episode/2niFpc437llS7Btn9UEH9D?si=oNGcYPNgTkukd4GqDfLFBA

(Dr. Sevtap Savas; Janine Taylor-Cutting)			
Mike Kehoe (PWLE)<br><br>(Dr. Sevtap Savas)	Newfoundland and Labrador, CANADA	Episode 14 – "You are not alone". Mike Kehoe shares his messages of hope for all folks out there	https://open.spotify.com/episode/72k610hyURQDs7F0ZjpARs?si=b1VQEKgqTYmSxBa5S2W2rw
Tracy Slaney (PWLE; L/A)<br><br>(Dr. Sevtap Savas)	Newfoundland and Labrador, CANADA	Episode 15 (special episode) – A heartwarming story of cancer advocacy and caregiving by Tracy Slaney	https://open.spotify.com/episode/1cLhelc423W32ALto74Rux?si=ZHTpgNgIQnCslXzAGRiKVw
Tracy Slaney (PWLE; L/A) and Heather Mulligan (L/A)<br><br>(Dr. Sevtap Savas)	Newfoundland and Labrador & Nova Scotia, CANADA	Episode 16 (special episode) – Role of advocacy in changing sick leave legislations in Atlantic Canada	https://open.spotify.com/episode/5QHjyk3z1x29Sksot1Sfvw?si=WUoJ-brWTxas0fm0NIkZKA
**SEASON II (12 podcast episodes)**			
Andrea Edwards (PWLE)<br><br>(Dr. Sevtap Savas)	Newfoundland and Labrador, CANADA	Episode 17– "Being a single mom with cancer is a real game changer"	https://open.spotify.com/episode/0VmKTvOlGZIAMPtYLO23iW?si=S4FVQFKpQVmE1eput7Ghow
Dr. Peter Barnes (HCP)	Newfoundland and Labrador, CANADA	Episode 18 – Grief as a transformative journey	https://open.spotify.com/episode/6dynAq8NBJWR4wItQXK9Va?si=qjj5LUf_Qdy6ztuS1JhFAw

(Dr. Sevtap Savas)			
Lianne Mantla-Look (PWLE; L/A)<br><br>(Dr. Sevtap Savas)	Northwest Territories, CANADA	Episode 19 – No backing down – Stomach cancer, self-advocacy, and living without a stomach	https://open.spotify.com/episode/6pulNrXfi21BFltzdqqINK?si=MdolLkdMTCabEhVxfqy1lw
Stephanie Howlett (PWLE)<br><br>(Dr. Sevtap Savas)	Newfoundland and Labrador, CANADA	Episode 20 – Experiences and perspectives by a queer cancer warrior	https://open.spotify.com/episode/3YjY6rdv8HVC4mCeAkmIBg?si=f0Zc8OvIR7O6ZCnjykiBpg
Dr. Don Desserud (PWLE)<br><br>(Dr. Sevtap Savas)	Prince Edward Islands, CANADA	Episode 21 – "But Don, all cancers are treatable"	https://open.spotify.com/episode/5tFvCmLMrGwUrbF35ZV5CY?si=J89J5xbqSR-6TR0vlbPdeA
Barry Stein (PWLE; L/A)<br><br>(Dr. Sevtap Savas; Tracy Slaney)	Quebec, CANADA	Episode 22 – "Cancer is preventable, treatable, beatable"	https://open.spotify.com/episode/5dAhukGj3TjcVj8sa7Eozk?si=flsgxyxCTROdgGZD4BbVRw
Lisa Ridgway (L/A)<br><br>(Dr. Sevtap Savas; Janine Taylor-Cutting)	British Columbia, CANADA	Episode 23 – Patient-oriented research from a patient partner & leader perspective	https://open.spotify.com/episode/4otZvfM2b59ENNKgUJbck2?si=Wj7fOflmQQOMSeTylUFs9w

Dr. Holly Etchegary (S)<br><br>(Janine Taylor-Cutting; Jason Wiseman)	Newfoundland and Labrador, CANADA	Episode 24 – Patient influencers of research	https://open.spotify.com/episode/0LHQNe9P0asPt0tMskfaPE?si=OTLS2TOZQeCc1h-1tx3awQ
Dr. Sheila Garland (S)<br><br>(Dr. Sevtap Savas)	Newfoundland and Labrador, CANADA	Episode 25 – Joy of reducing suffering through psychosocial oncology	https://open.spotify.com/episode/3w2ODyjaB09fezpUmUDzRD?si=_2Og8n9DTKSi_HWDiGrfLw
Dr. Lesa Dawson (HCP)<br><br>(Dr. Sevtap Savas)	British Columbia, CANADA	Episode 26 – Hereditary cancers	https://open.spotify.com/episode/1mkRb4SXzPWSO3p0X8Sm89?si=Qc6jyFq4SMWQzLLs75tvNw
Justin Andrews (L/A)<br><br>(Dr. Sevtap Savas	Newfoundland and Labrador, CANADA	Episode 27 – Experiencing cancer as a community member in the Big Land	https://open.spotify.com/episode/3PQzKKXMMxxbvyIZsDdcHX?si=nc_GLo0nTOSbpkalXjXVnQ
Dr. Melanie Seal (HCP)<br><br>(Dr. Sevtap Savas	Newfoundland and Labrador, CANADA	Episode 28 – Cancer care is team care	https://open.spotify.com/episode/1iPJMM1ng4aITceF8PqPaO?si=TBjNSX7DTjueMfyQ0mdQTQ

HCP: healthcare provider. L/A: leader/advocate. PWLE: people with lived experience (individuals affected by cancer directly or indirectly). S: scientist
